# Perioperative stroke in patients undergoing elective spinal surgery: a retrospective analysis using the Japanese diagnosis procedure combination database

**DOI:** 10.1186/s12891-015-0743-7

**Published:** 2015-10-02

**Authors:** Junichi Ohya, Hirotaka Chikuda, Takeshi Oichi, Hiromasa Horiguchi, Katsushi Takeshita, Sakae Tanaka, Hideo Yasunaga

**Affiliations:** Department of Orthopaedic Surgery, Faculty of Medicine, The University of Tokyo, Hongo 7-3-1, Bunkyo, Tokyo Japan; Department of Health Economics and Epidemiology Research, School of Public Health, The University of Tokyo, Hongo 7-3-1, Bunkyo, Tokyo Japan

**Keywords:** Perioperative stroke, Database, Spinal cord tumor, Hemorrhagic stroke, Ischemic stroke

## Abstract

**Background:**

Although a few studies on perioperative stroke following spinal surgery have been reported, differences in the incidence of perioperative stroke among various surgical procedures have not been determined. The purpose of this retrospective analysis was to investigate the incidence of perioperative stroke during hospitalization in patients undergoing elective spinal surgery, and to examine whether the incidence varied according to the surgical procedure.

**Methods:**

A retrospective analysis of data from the Diagnosis Procedure Combination database, a nationwide administrative impatient database in Japan, identified 167,106 patients who underwent elective spinal surgery during 2007–2012. Patient information extracted included age, sex, preoperative comorbidity, administration of blood transfusion, length of hospitalization, and type of hospital. Clinical outcomes included perioperative stroke during hospitalization, and in-hospital death.

**Results:**

The overall incidence of perioperative stroke was 0.22 % (371/167,106) during hospitalization. A logistic regression model fitted with a generalized estimating equation showed perioperative stroke was associated with advanced age, a history of cardiac disease, an academic institution, and resection of a spinal tumor. Patients who underwent resection of a spinal cord tumor (reference) had a higher risk of stroke compared with those undergoing discectomy (odds ratio (OR), 0.29; 95 % confidence interval (CI), 0.14–0.58; *p* = 0.001), decompression surgery (OR, 0.44; 95 % CI, 0.26–0.73; *p* = 0.001), or arthrodesis surgery (OR, 0.55; 95 % CI, 0.34–0.90); *p* = 0.02). Advanced age (≥80 years; OR, 5.66; 95 % CI, 3.10–10.34; *p* ≤ 0.001), history of cardiac disease (OR, 1.58; 95 % CI, 1.10–2.26; *p* = 0.01), diabetes (OR, 1.73; 95 % CI, 1.36–2.20; *p* ≤ 0.001), hypertension (OR, 1.53; 95 % CI, 1.18–1.98; *p* = 0.001), cervical spine surgery (OR, 1.44; 95 % CI, 1.09–1.90; *p* = 0.01), a teaching hospital (OR, 1.36; 95 % CI, 1.01–1.82; *p* = 0.04), and length of stay (OR, 1.008; 95 % CI, 1.005–1.010; *p* ≤ 0.001) were also risk factors for perioperative stroke.

**Conclusions:**

Perioperative stroke occurred in 0.22 % of patients undergoing spinal surgery. Resection of a spinal cord tumor was associated with increased risk of perioperative stroke as well as advanced age, comorbidities at admission, cervical spine surgery, surgery in a teaching hospital, and length of stay.

## Background

Perioperative stroke is a rare but devastating complication which can lead to prolonged hospital stay, persistent neurological impairment, and even death [1–3]. The majority of perioperative strokes are classified as ischemic stroke [4–6], though hemorrhagic strokes have also been reported. Known risk factors for perioperative stroke include advanced age, history of stroke, carotid stenosis, and atherosclerosis of the ascending aorta [1, 3, 5, 7–12]. The risk of perioperative stroke is also known to vary according to the type and complexity of the surgical procedure [3, 8, 10, 13–16].

Despite its significant impact on clinical outcomes, the available information on perioperative strokes following spinal surgery is limited, presumably owing to its rarity [17–21]. Although recent studies estimated the incidence to be 0.05–0.1 % [17, 18], the precise incidence remains unclear. In addition, the pathomechanism of perioperative stroke following spinal surgery remains largely unknown. Although a possible association between postoperative intracranial hemorrhage and cerebrospinal fluid (CSF) leakage subsequent to durotomy has been discussed in several case reports [17, 22–26], this remains to be established.

The purposes of this retrospective analysis using a Japanese national administrative database were: 1) to investigate the incidence of perioperative stroke during hospitalization in patients undergoing elective spinal surgery; and 2) to examine whether its incidence varies among different surgical procedures. We hypothesized that the procedures requiring durotomy (i.e., the resection of spinal tumor) would carry increased risk of perioperative stroke compared with the other procedures.

## Methods

### Data source

This study used data from the Japanese Diagnosis Procedure Combination (DPC) database [27–32], which includes administrative claims data and discharge abstract data from hospitals across Japan. All 82 university hospitals are obliged to contribute to this system, but adoption by community hospitals is voluntary. In 2011, approximately 50 % of all acute-care admissions in Japan were included in the DPC. The database includes the following information: unique identifier of the hospital and type of hospital (teaching or non-teaching); patient age and sex; main diagnoses; surgical procedures; comorbidities at admission and complications after admission recorded according to the International Classification of Diseases, 10^th^ Revision (ICD-10) codes; length of stay; and in-hospital death. Preexisting comorbidities at admission and complications occurring after admission are recorded separately in the DPC database. The anonymous nature of the data allowed the requirement for informed consent to be waived. Study approval was obtained from the Institutional Review Board of The University of Tokyo.

### Patient selection and data

We extracted patient data from the DPC for the period July 1, 2007 to March 31, 2012 for a total of 39 months: July 1 to December 31 of 2007–2010, January 1 to December 31 of 2011, and January 1 to March 31 of 2012 (DPC data were compiled in collaboration with the Ministry of Health, Labour and Welfare between July and December in 2007–2010 and have been compiled throughout the year since January 2011). We included patients aged 20 years or older who underwent elective spinal surgery (discectomy, decompression surgery, fusion surgery, resection of a spinal cord tumor). Patients requiring emergency admission and patients with a metastatic spinal tumor as the primary diagnosis were excluded from the study. The site of surgery was classified as cervical, thoracic, and lumbosacral. We assessed the patients’ clinical characteristics, including age, sex, comorbidities at admission, blood transfusion, length of stay, and type of hospital. Comorbidities at admission included a history of cardiac disease (angina, myocardial infarction, and heart failure), diabetes, hypertension, and a history of stroke.

### Outcomes

The outcomes included perioperative stroke (ICD-10 codes: I60-63) during hospitalization and in-hospital death. We further classified strokes into two categories: hemorrhagic (I60-62) and ischemic (I63).

### Statistical analyses

Continuous variables were compared using analyses of variance or the Kruskal-Wallis test, as appropriate, and proportions were compared using the Chi-square test or Fisher’s exact test. Multivariate logistic regression analysis was performed to determine factors associated with the occurrence of perioperative stroke. Because the data were clustered hierarchically (hospital and patient levels), we fitted the regression model with a generalized estimation equation to adjust for within-hospital clustering [33]. Variables that exhibited a significant difference in the univariate analysis were entered into a multivariate logistic regression analysis. The predefined significance for inclusion in the next step of the regression model was *p* ≤ 0.10. All statistical analysis were conducted using IBM SPSS version 19.0 (IBM Corp., Armonk, NY, USA). The threshold for significance was a *p*-value < 0.05.

## Results

A total of 167,106 patients were identified (98,445 male, 68,661 female). The mean age was 64.1 years (range, 20 to 101 years). The most common surgical procedure was decompression surgery (84,540 patients; 50.6 %), followed by arthrodesis surgery (52,784; 31.6 %), discectomy (24,595; 14.7 %), and resection of a spinal cord tumor (5187; 3.1 %). The most common surgical level was lumbar (109,669; 65.6 %). Table 1 presents the characteristics of the study participants according to the surgical procedures.

**Table 1:** Characteristics of the study population according to surgical procedures (*N* (%))

	Overall (*N* = 167,106)		Resection of spinal cord tumor (*N* = 5187)		Discectomy (*N* = 24,595)		Decompression surgery (*N* = 84,540)		Arthrodesis surgery (*N* = 52,784)		*p*-value^a^
Age, years; mean [SD]	64.1	[14.3]	57.1	[15.8]	50.2	[17.0]	68.2	[11.3]	64.8	[12.8]	<0.001
≤49	26,449	(15.8)	1607	(31.0)	12,395	(50.4)	5663	(6.7)	6784	(12.9)	<0.001
50–59	24,253	(14.5)	993	(19.1)	4088	(16.6)	10,937	(12.9)	8235	(15.6)	
60–69	45,030	(26.9)	1307	(25.2)	4269	(17.4)	23,881	(28.2)	15,573	(29.5)	
70–79	54,107	(32.4)	1011	(19.5)	2902	(11.8)	32,353	(38.3)	17,841	(33.8)	
≥80	17,267	(10.3)	269	(5.2)	941	(3.8)	11,706	(13.8)	4351	(8.2)	
Sex											
Male	98,445	(58.9)	2590	(49.9)	15,866	(64.5)	53,808	(63.6)	26,181	(49.6)	<0.001
Female	68,661	(41.1)	2597	(50.1)	8729	(35.5)	30,732	(36.4)	26,603	(50.4)	
Level of surgery											
Cervical	35,042	(21.0)	564	(10.9)	357	(1.5)	24,596	(29.1)	9525	(18.0)	<0.001
Thoracic	3582	(2.1)	465	(9.0)	345	(1.4)	822	(1.0)	1950	(3.7)	
Lumbosacral	10,9669	(65.6)	884	(17.0)	23,266	(94.6)	50,326	(59.5)	35,193	(66.7)	
Unspecified	18,813	(11.3)	3274	(63.1)	627	(2.5)	8796	(10.4)	6116	(11.6)	
History of cardiac disease	12,332	(7.4)	180	(3.5)	833	(3.4)	7356	(8.7)	3963	(7.5)	<0.001
Diabetes	27,052	(16.2)	453	(8.7)	2367	(9.6)	15,925	(18.8)	8307	(15.7)	<0.001
Hypertension	32,753	(19.6)	740	(14.3)	2511	(10.2)	18,947	(22.4)	10,555	(20.0)	<0.001
History of stroke	3807	(2.3)	70	(1.3)	217	(0.9)	2337	(2.8)	1183	(2.2)	<0.001
Hemodialysis	3340	(2.0)	65	(1.3)	147	(0.6)	1638	(1.9)	1490	(2.8)	<0.001
Blood transfusion	10,382	(6.2)	265	(5.1)	100	(0.4)	2091	(2.5)	7926	(15.0)	<0.001
Teaching institution	35,821	(21.4)	2891	(55.7)	3185	(12.9)	16,652	(19.7)	13,093	(24.8)	<0.001
Length of stay, days, median [IQR]	21	[16–30]	23	[17–33]	16	[12–22]	21	[16–28]	25	[19–37]	<0.001

^a^ *p*-value relating to comparison between four groups including resection of spinal cord tumor, discectomy, decompression surgery, and arthrodesis surgery

*SD* standard deviation, *IQR* interquartile range

Overall, perioperative stroke occurred in 371 patients (0.22 %) during hospitalization (Table 2). Of these, hemorrhagic stroke occurred in 53 patients (14.2 %) and ischemic stroke in 318 patients (85.7 %). Eighteen patients died of stroke and the case-fatality rate was 4.9 % (18/371).

**Table 2:** Perioperative stroke following elective spinal surgery

	Perioperative stroke (*N* (%))						
	Overall		*p*-value*	Hemorrhagic		Ischemic	
Total	371 (0.2)	(0.22)		53	(0.03)	318	(0.19)
Age, years			<0.001				
≤49	15	(0.06)		6	(0.02)	9	(0.03)
50–59	29	(0.12)		8	(0.03)	21	(0.09)
60–69	85	(0.19)		14	(0.03)	71	(0.16)
70–79	156	(0.29)		18	(0.03)	138	(0.26)
≥80	86	(0.50)		7	(0.04)	79	(0.46)
Sex			0.23				
Male	207	(0.21)		30	(0.03)	177	(0.18)
Female	164	(0.24)		23	(0.03)	141	(0.21)
Surgical procedures			<0.001				
Resection of spinal cord tumor	19	(0.37)		7	(0.13)	12	(0.23)
Discectomy	16	(0.07)		6	(0.02)	10	(0.04)
Decompression surgery	193	(0.23)		18	(0.02)	175	(0.21)
Arthrodesis surgery	143	(0.27)		22	(0.04)	121	(0.23)
Cervical spinal surgery	99	(0.28)	0.008	13	(0.04)	86	(0.25)
Others	272	(0.21)		40	(0.03)	232	(0.18)
History of cardiac disease	60	(0.49)	<0.001	6	(0.05)	54	(0.44)
No history	311	(0.20)		47	(0.03)	264	(0.17)
Diabetes	110	(0.41)	<0.001	9	(0.03)	101	(0.37)
No history	261	(0.19)		44	(0.03)	217	(0.15)
Hypertension	129	(0.39)	<0.001	17	(0.05)	112	(0.34)
No history	242	(0.18)		36	(0.03)	206	(0.15)
History of stroke	5	(0.13)	0.29	1	(0.03)	4	(0.11)
No history	366	(0.22)		52	(0.03)	314	(0.19)
Hemodialysis	15	(0.45)	0.01	4	(0.12)	11	(0.33)
No hemodialysis	356	(0.22)		49	(0.03)	307	(0.20)
Blood transfusion	52	(0.50)	<0.001	9	(0.09)	43	(0.41)
No transfusion	319	(0.20)		44	(0.03)	275	(0.18)
Teaching institution	107	(0.30)	0.001	17	(0.05)	90	(0.25)
Non-teaching institution	264	(0.20)		36	(0.03)	228	(0.17)

Table 3 shows the results of the multivariate logistic regression analysis for perioperative stroke. Perioperative stroke was associated with resection of a spinal tumor. Patients who underwent resection of a spinal cord tumor (reference) had a higher risk of stroke compared with those undergoing discectomy (odds ratio (OR), 0.29; 95 % confidence interval (CI), 0.14–0.58; *p* = 0.001), decompression surgery (OR, 0.44; 95 % CI, 0.26–0.73; *p* = 0.001), or arthrodesis surgery (OR, 0.55; 95 % CI, 0.34–0.90); *p* = 0.02). Advanced age (≥80 years; OR, 5.66; 95 % CI, 3.10–10.34; *p* ≤ 0.001), a history of cardiac disease (OR, 1.58; 95 % CI, 1.10–2.26; *p* = 0.01), diabetes (OR, 1.73; 95 % CI, 1.36–2.20; *p* ≤ 0.001), hypertension (OR, 1.53; 95 % CI, 1.18–1.98; *p* = 0.001), cervical spine surgery (OR, 1.44; 95 % CI, 1.09–1.90; *p* = 0.01), surgery in a teaching hospital (OR, 1.36; 95 % CI, 1.01–1.82; *p* = 0.04), and length of stay (OR, 1.008; 95 % CI, 1.005–1.010; *p* ≤ 0.001) were also identified as risk factors for perioperative stroke.

**Table 3:** Adjusted risk of perioperative stroke after elective spinal surgery

	Perioperative stroke		
	OR	95 % CI	*p*-value
Age			
≤49	Reference		
50–59	1.61	0.87–2.97	0.13
60–69	2.27	1.31–3.94	0.004
70–79	3.41	1.91–6.07	<0.001
≥80	5.66	3.10–10.34	<0.001
Sex			
Male	Reference		
Female	0.98	0.78–1.23	0.88
Surgical procedures			
Resection of a spinal cord tumor	Reference		
Discectomy	0.29	0.14–0.58	0.001
Decompression surgery	0.44	0.26–0.73	0.001
Arthrodesis surgery	0.55	0.34–0.90	0.02
Cervical spinal surgery	1.44	1.09–1.90	0.01
History of cardiac disease	1.58	1.10–2.26	0.01
Diabetes	1.73	1.36–2.20	<0.001
Hyper tension	1.53	1.18–1.98	0.001
Hemodialysis	1.51	0.88–2.59	0.13
Blood transfusion	1.23	0.83–1.82	0.30
Teaching institution	1.36	1.01–1.82	0.04
Length of stay (days)	1.01	1.01–1.01	<0.001

*OR* odds ratio, *CI* confidence interval

We further examined the proportion of hemorrhagic stroke among perioperative stroke in every surgical procedure. Among perioperative stroke, the proportion of hemorrhagic stroke following elective spinal surgery was 14.3 % (53/371). The proportion of hemorrhagic stroke among perioperative stroke following resection of a spinal tumor was 36.8 % (7/19), which was significantly higher compared with other procedures (vs 46/352, *p* = 0.01, Fisher’s exact test).

## Discussion

This study had three main findings. First, the incidence of perioperative stroke during hospitalization after elective spinal surgery was 0.22 %. Second, resection of a spinal cord tumor was associated with increased risk of perioperative stroke compared with other procedures. Finally, resection of a spinal cord tumor was associated with a higher incidence of hemorrhage stroke. The analysis of a nationwide database enabled us to investigate the incidence of this rare but devastating complication after elective spinal surgery and to evaluate its relevant risks.

Our findings were in line with previous reports, which showed the incidence of perioperative stroke following spinal surgery was 0.05–0.1 % [17, 18]. Smith et al. examined 108,419 procedures obtained from the Scoliosis Research Society Morbidity and Mortality database [19], which had a comparable sample size to that of our study. However, the exact incidence of postoperative stroke was not clear in that study, because the authors only reported fatal cases. To our knowledge, this study was the first report to compare the incidence of perioperative stroke following spinal surgery with different surgical procedures.

In the literature, perioperative stroke after surgery has been predominately classified as ischemic stroke [4–6]. Hemorrhagic stroke accounts for only 1 % of strokes occurring after coronary artery bypass grafting surgery [2]. In our study, we observed a higher percentage of hemorrhagic stroke following elective spinal surgery (14.3 %), which suggested that the pathomechanism of perioperative stroke varies according to the type of surgical procedure.

The current study showed that resection of a spinal cord tumor, requiring dural incision to access the tumor, had a higher risk of perioperative hemorrhagic stroke. Although intracranial hemorrhage after spinal surgery complicated by CSF leakage has been reported [17, 22–26], the mechanism of this complication remains speculative. In the presence of CSF leakage, the postulated pathophysiology in previous case reports was that an increase in transluminal venous pressure caused by intracranial hypotension from CSF loss may have resulted in blood vessel rupture [25]. Another possibility is that downward cerebellar sag causes stretching and occlusion of the bridging cerebellar veins [34]. Further investigations are needed to clarify the mechanism of intracranial hemorrhage after spinal surgery in order to prevent this catastrophic complication.

The results of this study showed that cervical spine surgery was also associated with the risk of perioperative stroke. Although cervical spine injury was reported to be a risk factor for blunt cerebrovascular injury [28, 35–37], a large-scale study on the relationship between elective spinal surgery at the cervical region and perioperative stroke has never been reported. However, a few reports regarding the impact of prolonged retraction during cervical anterior spinal surgery on carotid artery blood flow were reported [38, 39]. Moreover, Lunardini et al. showed, in their survey of 163,324 cervical spinal surgeries, that cerebellar infarction occurred in 5.5 % of patients with intraoperative vertebral artery injury [40]. Cervical spinal surgery, with a potential risk of intraoperative vertebral artery injury, may carry a higher risk for perioperative stroke.

There are a few limitations of this study. First, we were unable to investigate strokes occurring after discharge because of the lack of available data. However, according to OECD data, the national average length of hospital stay in Japan is 17.5 days, which is much longer than in other countries [41]. In Japan, hospitals often provide both early postoperative care and subsequent rehabilitation in a single hospitalization. Therefore, we believe that the follow-up period in this study, with a median of 21 days, was sufficient to detect the majority of strokes occurring after surgery, because most perioperative strokes reportedly occur within a few days after surgery. Indeed, approximately 45 % of perioperative strokes are reported within the first day after surgery [14, 42]. Second, the DPC database does not provide detailed clinical information such as severity of preoperative neurological symptoms, the level of fusion, and the presence of an incidental dural tear during spinal surgery. For instance, the DPC database does not include the “surgical level” as an entry field. In this analysis, we determined the surgical level based on the patients’ primary diagnosis (i.e., patients with lumbar spinal stenosis as their primary diagnosis were designated to have had lumbar spine surgery). In addition, we were unable to definitely differentiate intradural and extradural tumors from the DPC data. However, we excluded patients with metastatic spinal tumors, which have been reported to account for around 90 % of extradural spinal tumors [43, 44]. We believe the results of this study were unlikely to be affected by any unrecognized cases of extradural spinal tumor in this analysis. Third, the coded diagnoses may be less well validated than in a prospective survey because of possible misclassification or underreporting. However, we believe the rate of miscoding was relatively low because the diagnoses were recorded by the attending physicians. Finally, there may be some selection bias because of differences in participation rates between academic hospitals, which all contribute to the DPC database, and community hospitals, which only contribute voluntarily.

## Conclusions

This study revealed that perioperative stroke occurred in 0.22 % of patients who underwent elective spinal surgery. Resection of a spinal cord tumor was associated with a higher risk of perioperative stroke compared with other surgical procedures as well as advanced age, comorbidities at admission, cervical spine surgery, surgery in a teaching hospital, and length of stay. We believe the findings of this study provide useful information for a better understanding of the risk of perioperative stroke following elective spinal surgery.
